# Plasma levels and tissue expression of soluble TGFβrIII receptor in women with early-stage breast cancer and in healthy women: a prospective observational study

**DOI:** 10.1186/s12967-020-02659-4

**Published:** 2020-12-11

**Authors:** Lovorka Grgurevic, Ruder Novak, Vladimir Trkulja, Stela Hrkac, Grgur Salai, Josko Bilandzic, Lejla Ferhatovic Hamzic, Ivan Milas, Tiha Vucemilo, Melita Peric Balja, Karmen Bilic

**Affiliations:** 1 Center for Translational and Clinical Research, Department of Proteomics, School of Medicine, University of Zagreb, Zagreb, Croatia; 2 Department of Anatomy, Drago Perovic, School of Medicine, University of Zagreb, Drago Perovic, Zagreb, Croatia; 3 Department of Pharmacology, School of Medicine, University of Zagreb, Zagreb, Croatia; 4 University Hospital for Tumors, University Hospital Center Sestre Milosrdnice, Zagreb, Croatia; 5 Center for Proteomics, Center for Translational and Clinical Research, School of Medicine, University of Zagreb, Salata 11, Zagreb, Croatia

**Keywords:** Soluble TGFßrIII, Betaglycan, Breast cancer, Shedding

## Abstract

**Background:**

Mammary carcinogenesis is partly regulated by the transforming growth factor beta (TGFβ) signaling pathway. Its function in cancer progression and metastasis is highly dependent on disease stage, and it is likely modulated by the ratio of membrane-bound vs. soluble TGFβrIII (sTGFβrIII). In this prospective observational study, we assessed tissue expression and plasma levels of sTGFβrIII in healthy women, women with benign breast lesions and in early-stage breast cancer patients.

**Methods:**

In a preliminary study, plasma sTGFβrIII levels were determined in 13 healthy women (age 19–40 years) at different phases of the ovarian cycle, and in 15 patients (age 35–75 years) at different times of the day. The main study assessed plasma concentrations of sTGFβrIII in: (i) 158 healthy women in whom breast lesions were excluded; (ii) 65 women with benign breast lesions; (iii) 147 women with newly diagnosed breast cancer classified as American Joint Committee on Cancer (AJCC) stages 0 to IIB. Completers provided blood samples before surgery and at 10–30 and 160–180 days after surgery. Plasma sTGFβrIII concentrations were determined using an indirect ELISA kit. Part of the removed tissues underwent immunohistochemical (IHC) staining and analysis of tissue TGFβrIII expression.

**Results:**

There appeared no relevant variations in plasma sTGFßrIII levels at different times of the day or different ovarian cycle phases. Before surgery, breast cancer patients had somewhat higher sTGFβrIII than healthy women, or those with benign breast lesions (by 14.5 and 26 ng/mL, respectively), with a tendency of larger differences at higher age. This correlated with lower expression of TGFβrIII in breast cancer vs. healthy tissue samples. At 160–180 days after surgery, plasma sTGFβrIII levels in breast cancer patients declined by 23–26 ng/mL.

**Conclusions:**

Plasma sTGFβrIII levels do not seem to relevantly vary during the day or the ovarian cycle. The coinciding higher plasma levels in newly diagnosed cancer patients than in healthy subjects and lower TGFβrIII expression in the malignant than in healthy breast tissue suggest ectodomain shedding as a source of circulating sTGFβrIII. Decline in plasma levels after tumor removal supports such a view.

## Background

Breast neoplasms are the most common malignancies in women, and are the leading cause of cancer-related deaths in both developed and developing countries [1, 2]. Dysfunctional regulation of hormonal signaling pathways can drive cell proliferation and enable accumulation of genetic errors, which is important for initiation and progression of breast cancer. In recent years, there have been significant advances in breast cancer treatment, but timely diagnosis remains an important factor for patient survival. Several non-invasive procedures, like determining the expression levels of estrogen, progesterone and epidermal growth factor (EGF) receptors, are used for risk stratification and outcome prediction in breast cancer patients [3]. However, revealing the potential for metastatic changes is still largely based on clinical findings and radiologic examination, which often lack sensitivity, since they cannot detect micrometastatic disease. Detection of circulating tumor cells has recently been proposed as an independent early sign of breast cancer metastasis, but it lacks confirmation in prospective studies [4]. Neutrophil/lymphocyte ratio has also been investigated as a predictor of survival, although it has proven useful only in patients without metastatic disease [5].

Mammary carcinogenesis is partly regulated by the ubiquitous transforming growth factor beta (TGFß) signaling pathway, which is involved in a myriad of cellular functions, including development and homeostasis. The importance of preserved TGFβ signaling in cancer has been long recognized, since its loss, or even reduction, are correlated to poor disease prognosis [6]. However, its role in malignant transformation, tumor progression and metastasis is still poorly understood since it has a dichotomous function in different cancer types: although it can suppress tumorigenesis in early stages, it promotes tumor growth in late disease stages [7, 8]. This balance is likely fine-tuned through the availability of the TGFß receptor type III (TGFβrIII, betaglycan), whose role is to present ligands to the TGFβ type I and II receptors, leading to increased signaling through the canonical TGFβ pathway [6, 9–11]. This abundantly expressed co-receptor can act as a membrane bound, or a soluble effector. If its extracellular domain is released through ectodomain shedding, the soluble form of TGFβrIII (sTGFßrIII) is produced and it can be detected in the extracellular matrix, serum and milk [12]. Its function appears to be preserved as it can effectively bind all isoforms of TGFβ, bone morphogenetic proteins (BMPs), fibroblast growth factor (FGF) and inhibin [7, 9]. This proteolytic cleavage of TGFβrIII is highly regulated by matrix metalloproteinases and disintegrin metalloproteinase (ADAM) molecules [11]. The release of TGFßrIII reduces its membrane expression and decreases TGFβ signaling, since it prevents ligand presentation to the membrane-bound TGFβ receptors [13]. In breast cancer animal models it was shown that the downregulation of TGFβrIII/sTGFβrIII effectively dampens the host antitumor immune response, thus promoting cancer progression [14]. Accordingly, treatment with exogenous sTGFβrIII leads to an inhibition of tumor growth, metastasis and angiogenesis in breast cancer in vitro and in animal models [13, 14]. These findings are in line with tumor and metastasis promoting nature of TGF-β in the later stages of cancer progression [17].

Our previous smaller study [7], using an in-house developed polyclonal antibody, suggested lower plasma sTGFβrIII concentrations in women with American Joint Committee on Cancer (AJCC) stage 0-IIB breast carcinoma than in their healthy peers, and a trend towards increasing levels over 2 months after surgery in diseased women. In the present larger study, using a standardized commercial enzyme linked immunosorbent assay (ELISA) detection kit, we aimed to assess plasma sTGFβrIII concentrations in healthy women, women with benign breast lesions and women with AJCC stage 0-IIB breast cancer before surgical treatment and over a period of 6 months after surgery.

## Methods

**Fig. 1:**
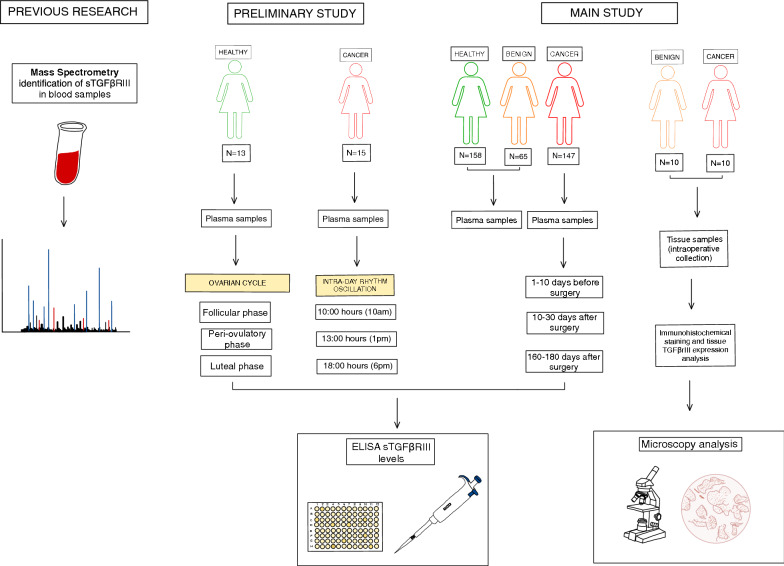
A schematic depiction of the study outline, subject groups and sample time points

*Study outline.* This prospective observational study was approved by the Ethics Committee of the University Hospital Center Sestre milosrdnice (EP-1402/18-3). All participants provided a signed informed consent. All blood samples were centrifuged to obtain platelet poor plasma and stored at −80 °C until analysis. In the preliminary part, plasma sTGFβrIII levels were determined in a smaller group of healthy women at different phases of the ovarian cycle, and in a smaller group of breast cancer patients at different times of the day (Fig. 1). The main study assessed plasma sTGFβrIII concentrations in: (i) healthy women in whom breast lesions were excluded during preventive check-ups; (ii) women with benign breast lesions, as identified by biopsy; (iii) women with breast cancer classified as AJCC stage 0 to IIB. Breast cancer patients were sampled at three time points: 1–10 days before surgery, at 10–30 and 160–180 days after surgery (Fig. 1). Part of the removed tissue underwent immunohistochemical (IHC) staining for TGFβrIII (Fig. 1).

### Preliminary evaluations

In order to evaluate the dependence of plasma sTGFßrIII levels on the menstrual cycle, 13 healthy women provided blood samples (between 09:00 and 11:00 a.m.) between days 2 and 8 of the ovarian cycle (follicular phase), between days 12 and 16 (mid-phase, peri-ovulatory) and between days 21 and 26–28 (luteal phase). To exclude the possibility of diurnal variation, 15 hospitalized women with diagnosed breast cancer (before treatment) provided blood samples at 10:00, 13:00 and 18:00 h on the same day.

### Patient management

A common inclusion criterion was signed informed consent. Women diagnosed with breast cancer (“cases“) AJCC stage 0-IIB were surgically treated. Primary surgical procedures included: mastectomy (with or without primary reconstruction), breast-conserving surgery, sentinel lymph node biopsy, or axillary lymph node dissection. Indication for the type of primary surgery was determined by a multidisciplinary team depending on the size of the tumor, localization, its biology and the patient’s preference. Therapeutic goals included complete resection of the primary tumor, with negative margins to reduce the risk of local recurrences, and pathologic staging of the tumor and axillary lymph nodes to provide necessary prognostic information. Based on patient’s age and cardiovascular status, and in line with the tumor characteristics, adjuvant chemotherapeutic protocols were individually selected and commenced between the 4th and 6th postoperative week. Premenopausal women with hormone receptor-positive tumors were treated with tamoxifen, whereas postmenopausal women were treated with aromatase inhibitors. IHC was used to test for human epidermal growth factor receptor 2 (HER2)-positive tumors and borderline-significant specimens were further analyzed by the fluorescence in situ hybridization (FISH). Patients with HER2-positive tumors were treated with trastuzumab for one year. Locoregional irradiation was administered in women who met the radiation criteria. Patient follow-up included in this study occurred at two time points: 10–30 days and 160–180 days after surgery, i.e. tumor mass removal.

### Detection of sTGFßrIII

Blood samples for sTGFßrIII plasma measurements in the main part of the study were taken during a.m. All procedures and evaluation of the results were conducted by researchers blinded to clinical and pathological patient data. sTGFßrIII in plasma was detected using an indirect ELISA kit (Human TGF-beta RIII DuoSet DY242, R&D, Minneapolis, MN), according to manufacturer’s instructions. Results were obtained with a plate reader (Molecular Devices–SpectraMax i3x) at 540 nm. All samples and standards were analyzed in duplicates and samples with an individual coefficient of variation (CV) greater than 25% were retested in duplicates. Tissue sTGFβrIII expression levels in benign and malignant breast lesions (N = 10 randomly chosen samples per group) were determined on formalin-fixed paraffin-embedded tissue sections by IHC. Selected tissues were cut in 3–4 μm sections, mounted on slides, and dried at 60 °C for 60 min. Dewaxing and target heat retrieval were performed simultaneously in automated PTLink (Dako) for 20 min at 97 °C in Target retrieval solution (3 in 1), pH 9.0 (S2367; Dako, Glostrup, Denmark). After blocking peroxidase with 5% hydrogen peroxide for 5 min, sections were incubated with a primary murine monoclonal antibody against TGFβrIII, (clone A4, sc-74511, Santa Cruz Biotechnology, USA) at a dilution of 1:50, at room temperature for 60 min. Thereafter, a secondary antibody conjugated to horseradish peroxidase (EnVision Flex/HRP high pH; Dako, Denmark) was applied for 50 min. Finally, sections were incubated with 3,3′-diaminobenzidine chromogen, contrasted with hematoxylin, and cover-slipped. Benign lesions included fibroadenomas, tubular adenomas, adenosis, usual ductal hyperplasia, and mastitis. Malignant lesions included in situ and invasive cancers. Most invasive cancers were ductal invasive carcinoma (no special type) and one case was a lobular invasive cancer. Adenosis as a benign breast condition served as a positive control, whereas tissue sections stained with murine immunoglobulin G instead of a primary antibody served as a secondary antibody only control. Stained slides were analyzed using an optical microscope (Zeiss Axiostar plus, magnification range 20X and 40X). IHC evaluation was performed by two investigators blinded to clinical and pathologic data (LG, GS) and reconfirmed by a second evaluation by a board-certified pathologist (MPB) blinded to the interpretations of the first set of evaluations. Immunostaining results were compared, and discrepancies were reviewed. There was significant agreement between the three observers (98% correspondence); thus, the pathologist’s scores are presented.

### Data analysis

In preliminary evaluations, ln-transformed plasma sTGFßrIII concentrations were analyzed by fitting generalized linear mixed models with time (ovarian cycle phase or time of the day) and age as fixed effects. Plasma concentrations in healthy women, women with benign lesions and women with breast cancer before surgery were compared by fitting a generalized linear model with health status, age and health status*age interaction, and effects were expressed as adjusted mean differences. Plasma sTGFßrIII concentrations in women with breast cancer determined at different time points after surgery were compared to values before surgery by fitting generalized linear mixed models with time point, age, time point*age interaction and AJCC stage as fixed effects. All confidence intervals and P-values were adjusted for multiplicity by the simulation method. We used SAS for Windows 9.4 (SAS Inc., Cary, NC), procedure GLIMMIX.

## Results

### Preliminary study

**Fig. 2:**
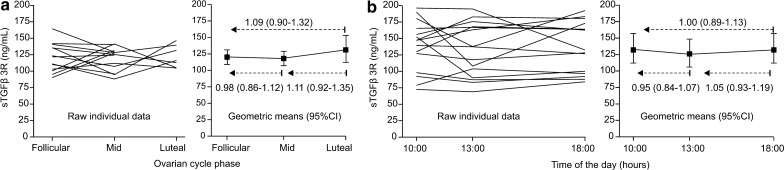
Preliminary study. **a** Plasma sTGFβrIII expression levels at different parts of the ovarian cycle in 13 healthy women aged 19-40 years. **b** Plasma sTGFβrIII expression levels at different times of the day in 15 hospitalized women with breast cancer AJCC stage 0-IIB (prior to surgery), aged 35-75 years

Plasma sTGFβrIII expression levels appeared similar at different ovarian cycle phases in healthy women (geometric means ratios (GMRs), at mid- and luteal phase vs. follicular phase closely around 1.0; Fig. 2a) and at different times during the day in women with breast cancer (GMRs at 13:00 and 18:00 h vs. 10:00 h closely around 1.0; Fig. 2b).

### Plasma sTGFβrIII levels in women with breast cancer, benign lesions and in healthy women

**Fig. 3:**
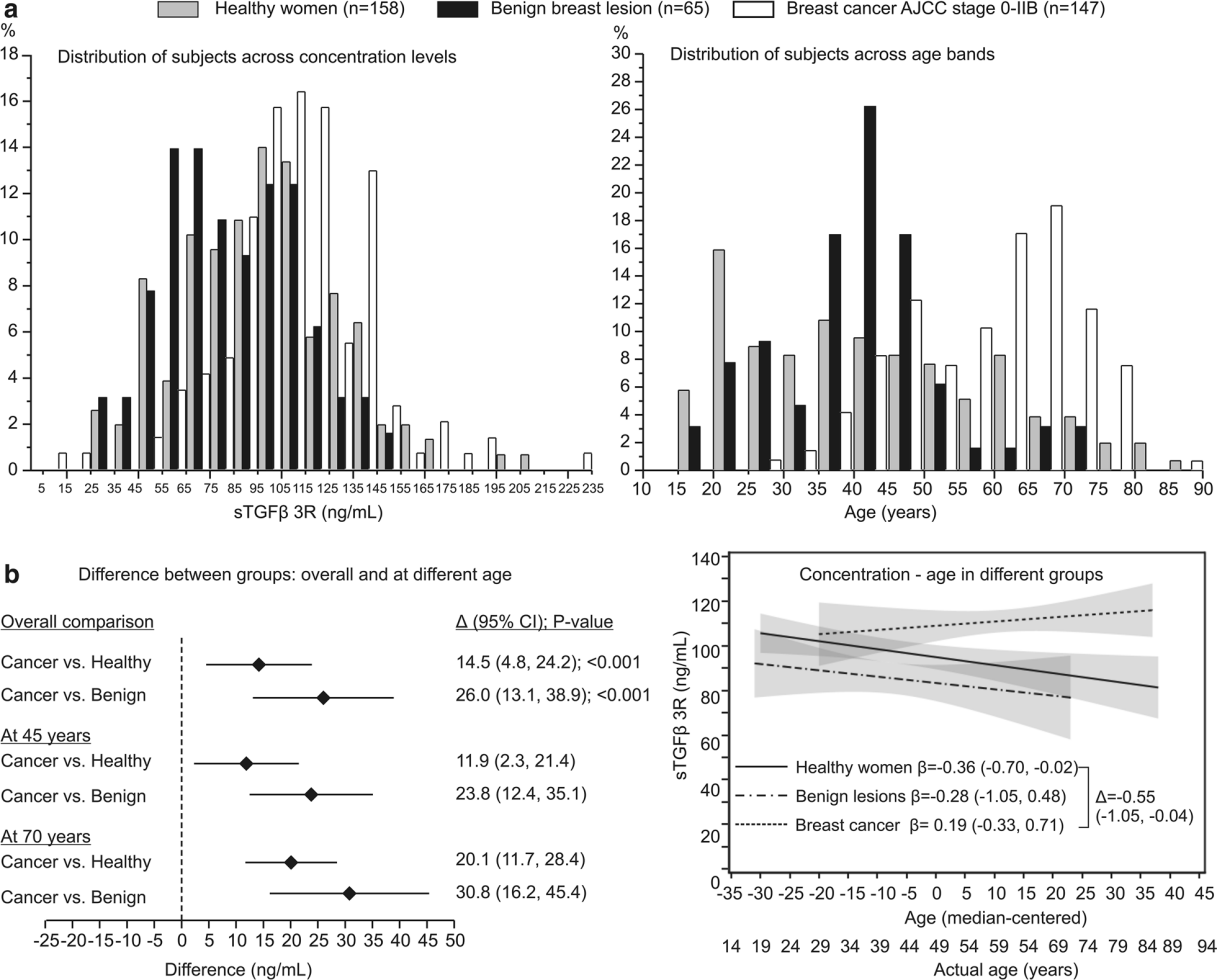
**a** Distribution of healthy women, women with benign lesions and of breast cancer patients in respect to plasma sTGFβrIII expression levels and age. **b** Age-adjusted differences in plasma sTGFβrIII expression levels between subject groups (left) and regression coefficients of plasma sTGFβrIII expression level on age across the three subject groups (right: depicted are regression coefficients with 95% confidence interval (CI) and a difference in coefficient between healthy women and breast cancer patients). These effects were obtained in a general linear model with group, age and group*age interaction effects

A total of 158 healthy women, 65 women with benign lesions and 147 women with AJCC stage 0-IIB breast cancer (13 stage 0 [carcinoma in situ], 47 stage IA, 33 stage IB, 45 stage IIA and 9 stage IIB) were enrolled in the main part of the study. On average, women with breast cancer were older and had somewhat higher sTGFβrIII plasma levels (similarly across tumor stages) taken before surgery than healthy women and women with benign breast lesions (Table 1). Figure 3a illustrates the “shift” in age and concentration distributions. With adjustment for age, sTGFβrIII plasma concentrations were higher in women with breast cancer than in healthy women (by around 14.5 ng/mL) and in women with benign lesions (by around 26 ng/mL) (Fig. 3b) with a tendency of larger differences at higher age (Fig. 3b); conversely, in healthy women and women with benign lesions, concentrations tended to decrease with older age, but no such trend was apparent in women with breast cancer (Fig. 3b).

**Table 1:** Age and sTGFβrIII plasma concentrations in included women. Data are median (quartiles; range) or mean ± SD (range)

	Women without breast carcinoma		Women with breast carcinoma		
	Healthy women	Benign lesions	All	AJCC stage 0-I	AJCC stage IIA-B
N	158	65	147	93	54
Age (years)	41 (37-56; 19-87)	42 (35-48; 18-72)	63 (50-69; 20-86)	63 (50-70; 31-79)	63 (50-69; 29-86)
sTGFβrIII (ng/mL)	97 ± 33 (29-215)	85 ± 28 (32-153)	111 ± 31 (6.3-230)	110 ± 33 (6.3-230)	113 ± 27 (52-190)

*AJCC* American Joint Committee on Cancer

### Plasma sTGFβrIII levels in women with breast cancer after surgery

**Fig. 4:**
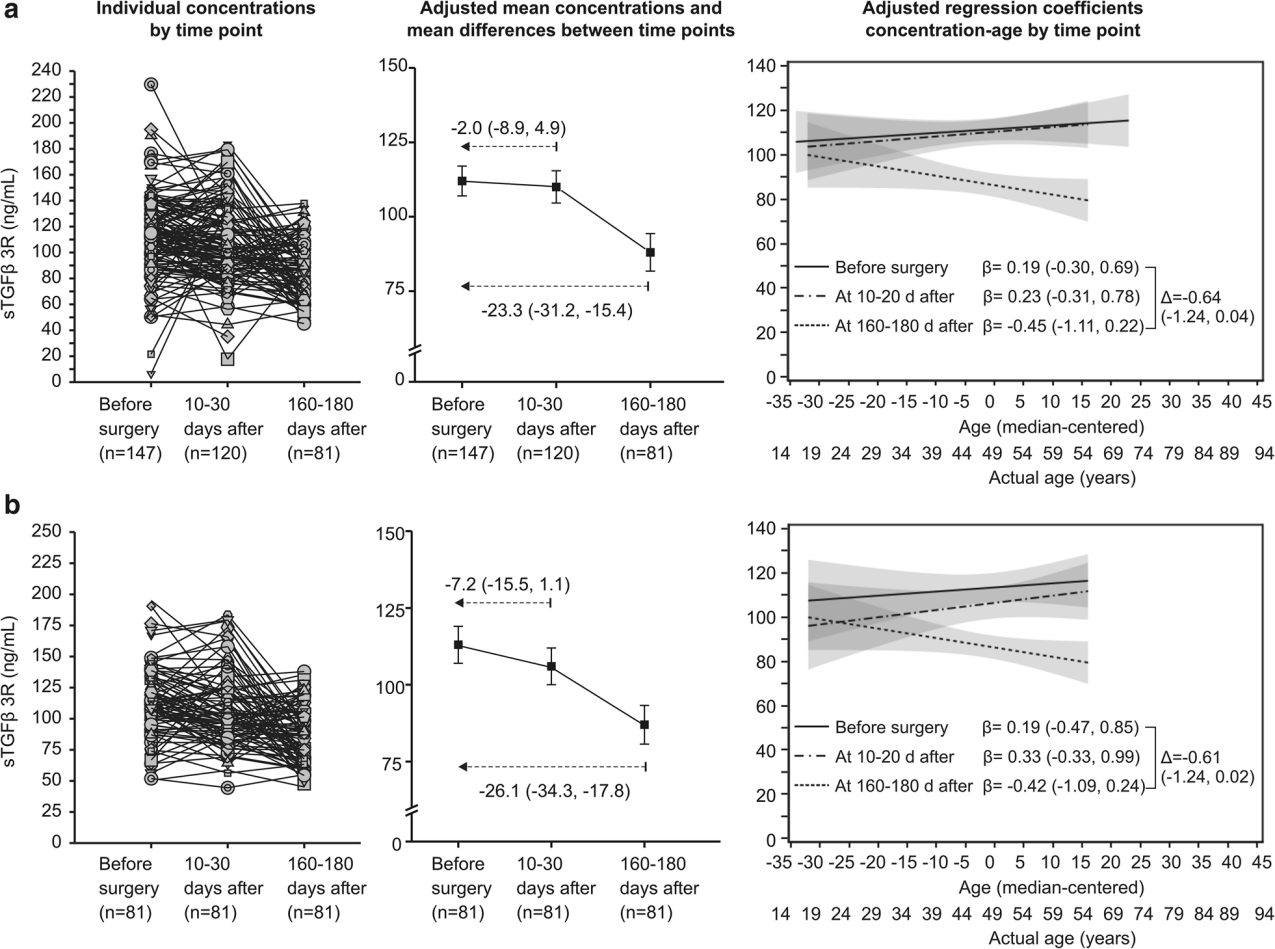
Plasma sTGFβrIII expression levels in breast cancer patients over time. **a** All available data. **b** Data for 81 patients that contributed blood samples at all time-points

Of the 147 women with AJCC stage 0-IIB breast carcinoma, all with pre-surgery determined sTGFβrIII levels, 120 provided blood samples at 10-30 days after surgery and 81 provided the samples at all three time points. “Completers” (n = 81) were comparable to “non-completers” (n = 66) in respect to age [median 63 (range 31–79) years vs. median 62 (range 29–86) years] and AJCC stages (67.9% stage 0-I vs. 57.6% stage 0-I and 42.4% stage IIA-IIB). Individual data for all women and for the subset of “completers” indicated a trend towards reduction of sTGFβrIII concentrations over time (Fig. 4). With adjustment for age, AJCC stage and time*age interaction, differences between 10 and 30 days after surgery and values before surgery were minor in both patient subsets, but values at 160–180 days after surgery were clearly lower (by around 23–26 ng/mL) vs. the values before surgery (Fig. 4). Moreover, there appeared to be no association between age and plasma sTGFβrIII concentration at 10-30 days after surgery (i.e., the coefficients were similar to those observed before surgery), while at 160-180 days after surgery, there was a tendency of lower concentrations with older age (Fig. 4), just as observed in healthy women (Fig. 3). None of the women died or experienced disease recurrence during the observed period, and none died over a period of up to 3 years (last patient’s last sample) since the recruitment of the first patient.

### TGFβrIII expression in breast cancer tissue

**Fig. 5:**
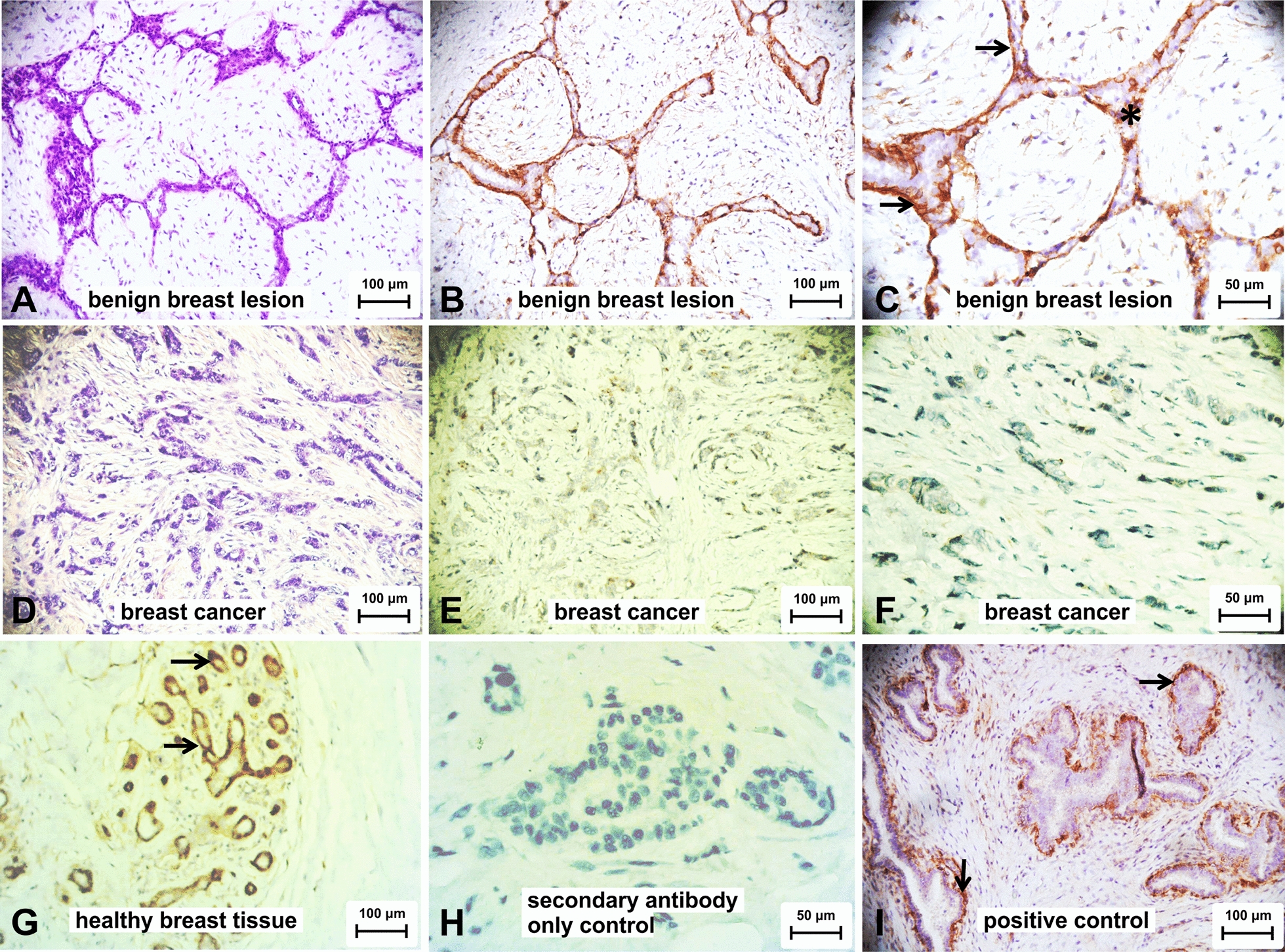
Loss of TGFβrIII protein in breast cancer. TGFβrIII expression was analyzed by immunohistochemistry (IHC) using anti TGFβrIII (sc-74511) and chromogenic detection. IHC staining was scored on a 0 to 3 scale by a board-certified pathologist, 0 corresponding to no staining and 3 to high staining levels. H&E staining of benign breast tissue lesion (**a**) and breast cancer (**d**). Representative staining of TGFβrIII in a benign breast lesion (**b**, 20x; **c** 40x), with positive epithelial (asterisk) and myoepithelial cells (arrows). Similar results were obtained in healthy breast tissue, obtained as an internal control from each sample (**g**, 20x). Staining of TGFβrIII in breast cancer tissues (**e**, 20x; **f**, 40x) showed no reaction in the majority of analyzed samples. Secondary antibody only control (**h**); positive tissue control, benign breast tissue lesion (**i**). Note the significant decrease in staining intensity in breast cancer (**e**, **f**) in comparison to healthy tissues (**g**) and benign lesion of breast tissue (**b**, **c**)

Prior to IHC staining, all hematoxylin–eosin-stained slides were examined to determine a sample that, in addition to the tumor/benign lesion, contained normal breast tissue as an internal control. Breast ducts and lobules in benign lesions showed clear expression of TGFßrIII, which was strongest on the membranes of epithelial and especially in myoepithelial cells. TGFßrIII expression was strong in 2, moderate in 6, and weak in 2 patients (Fig. 5b, c).

**Fig. 6:**
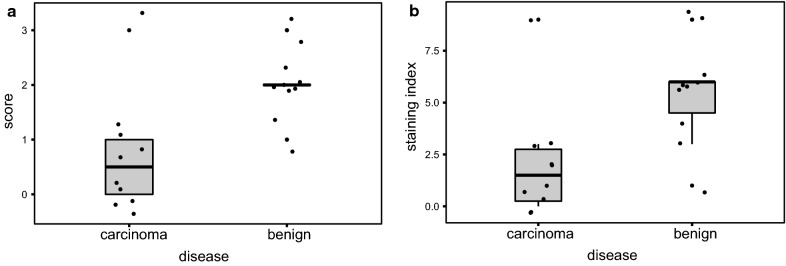
TGFβrIII immunohistochemistry quantification results: scores (**a**) and staining indices (**b**). Dots are individual values, horizontal lines depict medians, boxes are quartiles and bars are inner fences

TGFßrIII was not detected in 6 of the analyzed malignant cases and 4 showed a weak, albeit detectable membrane staining (Fig. 5e, f). A single case had moderate to strong membrane expression, and a patient with lobular invasive carcinoma had a mild cytoplasmic TGFßrIII staining. Distribution of TGFβrIII scores and staining index values for patients with breast carcinoma or benign breast lesions is shown in Fig. 6.

## Discussion

The multifunctional TGFß pathway affects the disease course of breast cancer patients, so it is important to understand the fine mechanisms of its regulation [18]. When TGFß binds to its receptors (TGFßrI and II), their activation seems to be dependent on a Type III co-receptor membrane-bound molecule. This co-receptor can nevertheless be rapidly cleaved and released into extracellular space, thus competing for available TGFß molecules and leaving the receptors “empty handed”. It was previously shown that dysregulation of TGFßrIII is an early event in breast cancer development, however this co-receptor can also have an anti-tumor effect [12]. The crucial event is the solubilization (release) of the membrane form, which competes for TGFβ ligands, and thus inhibits TGFβ-mediated tumor progression [16]. The ratio of membrane bound vs. soluble form of this co-receptor was also shown to regulate activity of other important breast cancer mediators, like the BMP-family proteins [19]. Therefore, TGFßrIII can exert different downstream effects depending on its expression levels and cellular localization.

We previously conducted a small-sample study that revealed an increase in plasma sTGFßrIII levels in early-stage breast carcinoma patients two months post breast cancer surgery [7]. Different research groups have since published contradictory results, which prompted a further investigation with a larger number of patients using a validated commercial ELISA kit and extensive controls to exclude possible physiologic oscillations of sTGFßRIII, unrelated to the disease [6, 19]. It is well known that cytokine production can be influenced by sex hormones and diurnal variation. We have thus excluded their influence on plasma sTGFßrIII in a preliminary study, by sampling patients at different menstrual-cycle time points and different times of day. In the main study, plasma levels of sTGFβrIII in breast cancer patients were monitored before and after surgery and compared to benign breast lesions and healthy controls. Furthermore, we analyzed the tissue expression of TGFßrIII in samples of randomly selected women with breast cancer and benign breast disease in order to correlate circulating to membrane-bound co-receptor levels. In contrast to the pilot study, our repeated results were in line with several other reports, which can be accounted for by the use of optimized detection antibodies and a larger sample size. Present data suggest two potentially important conclusions: i) plasma levels of sTGFβrIII do not seem to be dependent on menstrual cycle or diurnal variations; ii) reduced TGFβrIII expression in breast cancer tissue could result from ectodomain shedding.

The present data indicate higher plasma sTGFβrIII levels in (early-stage) breast cancer patients than in healthy women and women with benign breast lesions (which, in turn, are comparable to each other in this respect). The observed differences appeared greatest in women over 50 years of age. At 160–180 days after surgery, i.e., tumor mass removal, plasma levels of sTGFßrIII declined towards values seen in healthy women. Another indicator of “normalization” (post-tumor removal) is the regain of the association between older age and lower sTGFßrIII levels seen in healthy women, but not in breast cancer patients before surgery. Breast cancer screening programs mostly target postmenopausal women, since the worldwide disease incidence peaks around the age of 60, with a sharp incline beginning at 40 years of age [20, 21]. Therefore, the use of sTGFβrIII as a potential early plasma biomarker of breast cancer should be further explored. As breast cancer patients tend to be of older age, this assay could potentially complement mammography screenings and help discern diagnostically problematic lesions in postmenopausal women. It also seems feasible to extend the research to evaluate whether long-term follow-up with occasional measurements of plasma sTGFβrIII levels could be used for early detection of disease relapse.

However, it is always a challenge to distinguish metabolic events that are pathologically significant from ones that are a mere protein turnover mechanism, unrelated to carcinogenesis. The role of TGFßrIII in this spectrum still needs to be clarified: is it part of an anti-cancer mechanism that averts TGFß-related carcinogenesis or a consequence of the disease that can nevertheless be used as an indicator of disease occurrence, progression, or the efficacy of therapeutic interventions.

**Fig. 7:**
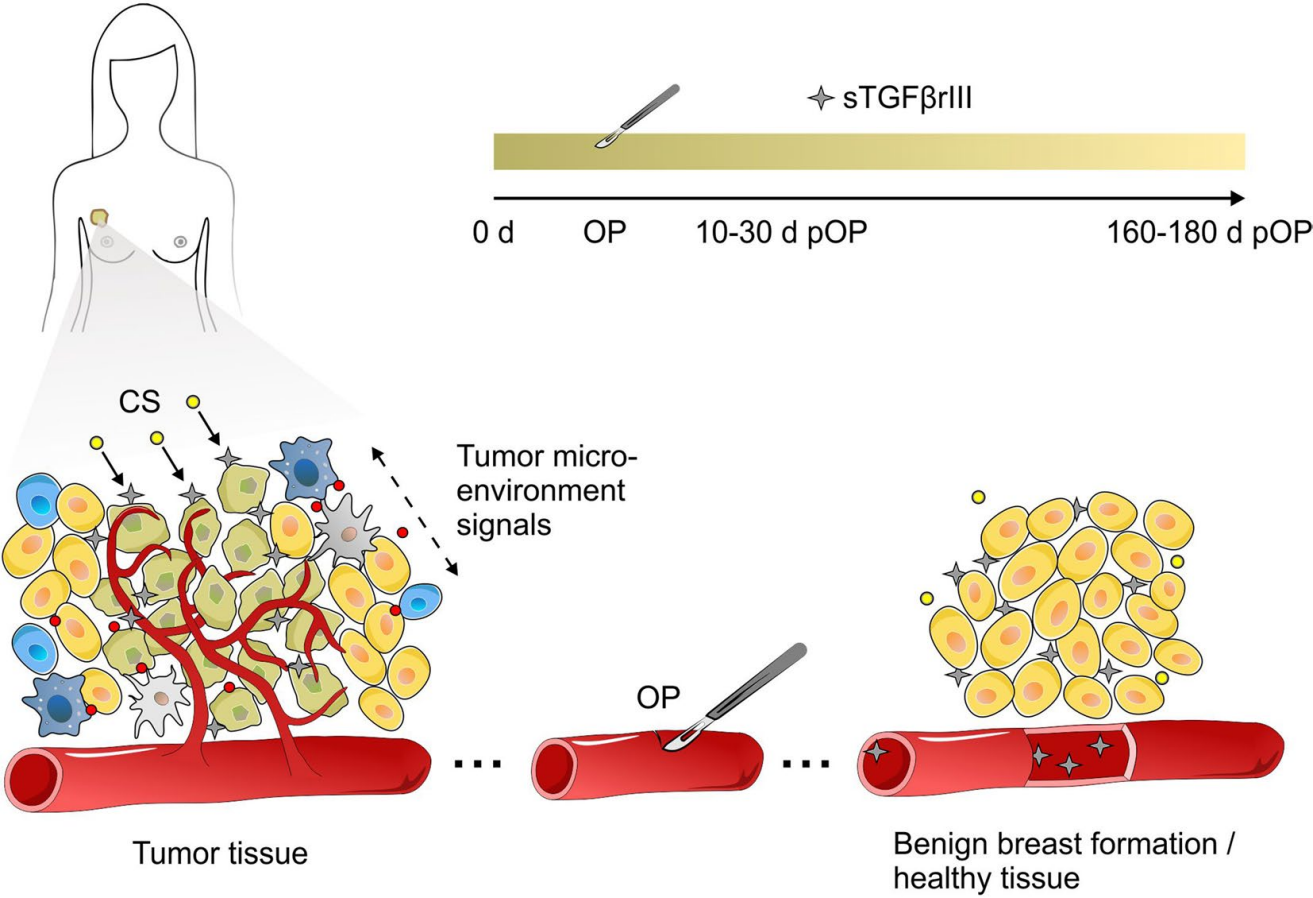
Proposed scheme for tumor microenvironment influence on TGFβrIII shedding from cell surface and modulation of its concentration in bodily fluids. Tumor cells recruit stromal, immune and vascular cells by secreting stimulatory growth factors, chemokines and cytokines. These cells release growth-promoting signals, which have a role in remodeling of tumor structure microenvironment and catalytic sheddases (CS) activity. This results in increased release of sTGFβrIII to the surrounding tissue and bodily fluids. After surgical tumor removal and the stabilization of the tissue CS activity (4–6 months), sTGFβrIII levels are normalized to a level found in healthy and benign breast formations

The origin of plasma sTGFßrIII is under debate. In complex tumor environments numerous soluble and membrane bound proteases (sheddases) can release a broad array of substrates into circulation [23]. In 2019, Huang et al. suggested that fibroblasts, immune and endothelial cells from the tumor environment could be the source of plasma sTGFβrIII [6]. The present data support such a view, as we observed a significant drop in plasma sTGFßrIII levels when the tumor tissue was surgically removed. Moreover, IHC findings revealed that lower tissue TGFβrIII expression levels in breast cancer patients than in healthy women and patients with benign breast changes. Since sTGFβRIII is readily detectable in plasma of healthy people and in patients with benign breast changes, we can assume that the co-receptor is continuously released at low levels. The reduction of TGFßrIII tissue expression was confirmed in most human cancers, and was also correlated to disease progression and prognosis [17–19, 23–25]. This simultaneous exchange seems to result from ectodomain shedding that releases soluble receptor forms from tissues into circulation. This process is closely related to the etiology of malignant disease and could be a physiologic response used to neutralize the effects of TGFβ molecules on tumor cell invasion, motility and migration [12, 26] (Fig. 7).

The present study is limited by still a modest sample of patients recruited at a single center and inclusion of only early-stage patients (AJCC 0-IIB) with favorable outcomes. The latter was a planned decision as it enabled an insight into sTGFßrIII levels before and after a (presumably) total tumor mass removal. A further limitation is a rather high drop-out of patients at repeated post-surgery blood samplings (patients chose not to participate). However, a similar trend was observed when all available data at each time-point were considered and when only “completers” were considered. Furthermore, the two subsets (“completers” and “drop-outs”) appeared similar in age and AJCC stage. Finally, the follow-up period (3 years for clinical outcomes and 6 months for plasma concentrations) was also limited, but it detected a trend that justifies a larger study focused on postmenopausal women. This (sub)population is at the highest risk from this disease, and it is suitable to further evaluate the usability of plasma sTGFβRIII as a potential complementary tool in breast cancer diagnosis and disease monitoring.

## Conclusions

In this prospective study we found that plasma sTGFßrIII levels in newly diagnosed early-stage (AJCC 0-IIB) breast cancer patients are higher than in healthy women or women with benign breast lesions. These findings are in correlation with lower tissue expression of TGFßrIII in malignant lesions vs. healthy breast tissue and implicate ectodomain shedding as a possible source of circulating sTGFßrIII. Furthermore, the difference between cancer and non-cancer participants appears to be greater in women over 50 years of age: we observed that in the non-cancer participants (but not in cancer patients) sTGFßrIII levels decline with age. Moreover, after surgical tumor removal, plasma sTGFßrIII levels decline towards levels seen in healthy women, and the association between older age and lower levels is thus re-gained. Additionally, plasma sTGFßrIII levels do not appear to change significantly throughout the ovarian cycle or time of day.

## Data Availability

Study data is available upon reasonable request.

## Abbreviations

ADAM: Disintegrin and metalloproteinase domain-containing protein
AJCC: American Joint Committee on Cancer
BMP: Bone morphogenetic protein
CI: Confidence interval
CS: Catalytic sheddase
CV: Coefficient of variation
EGF: Epidermal growth factor
ELISA: Enzyme linked immunosorbent assay
FGF: Fibroblast growth factor
FISH: Fluorescence in situ
GMR: Geometric means ratio
HER2: Human epidermal growth factor receptor 2
HRP: Horseradish peroxidase
IHC: Immunohistochemistry
SD: Standard deviation
sTGFβrIII: Soluble type III transforming growth factor receptor
TGFβ: Transforming growth factor β
TGFβrIII: Type III transforming growth factor receptor
